# Optical Imaging in Breast Cancer Diagnosis: The Next Evolution

**DOI:** 10.1155/2012/863747

**Published:** 2012-12-04

**Authors:** Michel Herranz, Alvaro Ruibal

**Affiliations:** ^1^Molecular Imaging Program, PET Radiopharmacy Unit, Molecular Imaging Group, IDIS, GALARIA-SERGAS, University Hospital Complex, Travesía de Choupana s/n., 15706 Santiago de Compostela, Spain; ^2^Nuclear Medicine Service, Medicine Faculty, Molecular Imaging Group, IDIS, University Hospital Complex, Travesía de Choupana s/n., 15706 Santiago de Compostela, Spain

## Abstract

Breast cancer is one of the most common cancers among the population of the Western world. Diagnostic methods include mammography, ultrasound, and magnetic resonance; meanwhile, nuclear medicine techniques have a secondary role, being useful in regional assessment and therapy followup. Optical imaging is a very promising imaging technique that uses near-infrared light to assess optical properties of tissues and is expected to play an important role in breast cancer detection. Optical breast imaging can be performed by intrinsic breast tissue contrast alone (hemoglobin, water, and lipid content) or with the use of exogenous fluorescent probes that target specific molecules for breast cancer. Major advantages of optical imaging are that it does not use any radioactive components, very high sensitivity, relatively inexpensive, easily accessible, and the potential to be combined in a multimodal approach with other technologies such as mammography, ultrasound, MRI, and positron emission tomography. Moreover, optical imaging agents could, potentially, be used as “theranostics,” combining the process of diagnosis and therapy.

## 1. Introduction

Breast cancer is a major global health problem. In 2007, an estimated 1.3 million new cases of invasive breast cancer will be diagnosed and about 465,000 women are expected to die from this disease worldwide [1]. Approximately one in nine women will develop breast cancer in their lifetime, and of these cancers, approximately 30% will be lethal [2].

Breast cancer is one clear example of excellent survival statistics when early-stage disease is treated using current therapies. Currently, numerous clinical methods are used in breast cancer screening and diagnosis [3]. The most effective screening technique at this time is X-ray mammography. The overall sensitivity of X-ray mammography for breast cancer detection is moderate (75%), and even more reduced in women with dense breasts: 62% [4, 5]. X-ray mammography has a 22% false positive rate in women under 50 [6]. The method cannot accurately distinguish between benign and malignant tumors [7]. 

Techniques such as magnetic resonance imaging (MRI) and ultrasound are sometimes used in addition to X-ray mammography, but have limitations such as high cost, low throughput, limited specificity (MRI), and low sensitivity (ultrasound). Thus, there is still a need to detect cancers earlier for treatment [4, 5], missed by mammography [8], and to add specificity to the procedures, since the majority of invasive follow-up procedures (e.g., surgical biopsies) are performed on normal or benign tissue.

New methods are being investigated to bridge the current gap in clinical utility. Examples of such experimental techniques are elastography, tomosynthesis, dedicated computed tomography (CT) and positron emission tomography (PET), photoacoustic (or optoacoustic) imaging, and optical imaging.

## 2. Breast Cancer Imaging

Breast imaging is largely indicated for detection, diagnosis, and clinical management of breast cancer. Commonly used imaging modalities include mammography, ultrasonography, magnetic resonance imaging (MRI), scintimammography, single photon emission computed tomography (SPECT) and positron emission tomography (PET).

### 2.1. Mammography

Mammography is, essentially, the only widely used Imaging modality for breast cancer screening. Several large randomized clinical trials have shown that mammography reduces mortality from breast cancer [9–12]. Extensive investigations on radiation dose to the breast and its dependence on breast composition, breast thickness, and X-ray spectral characteristics have been documented [13, 14]. Calcifications, or soft-tissue hardening with calcium deposits, are especially important. They are often an early sign of breast cancer, especially if the calcifications are small (microcalcifications) or irregularly shaped. The study does have some limitations. Imaging is more difficult with breasts that are dense or breasts in younger women. Breasts with implants or significant surgical scars are also difficult to visualize on mammography. 

### 2.2. Ultrasonography

The role of ultrasound in breast imaging has been largely limited to applications such as distinguishing between cystic versus solid masses, evaluation of palpable masses, and for needle core biopsy. In recent years, the number of indications has been greatly expanded and breast ultrasonography is now an essential modality in breast imaging. Colour Doppler and more recently sensitive power Doppler ultrasound has been used for further evaluation of the breast. In 2002, Kolb et al. published a fundamental article that showed improved sensitivity (97% versus 74%) when adjunctively used with mammography compared to physical examination with mammography [15]. However, there was also a substantial decrease in positive predictive value with mammography plus ultrasound (11.2%) compared to mammography (22.6%) alone. Ultrasound has become a valuable tool to use with mammograms because it is widely available, noninvasive, and less costs than other options. But, ultrasound test value depends on the operator's level of skill and experience.

### 2.3. Magnetic Resonance Imaging

Independent clinical trials for women at high risk of hereditary breast cancer indicate increased sensitivity with breast MRI than mammography but with variable specificity. Dynamic contrast enhanced MRI (DCE-MRI) is a very important tool for detection, diagnosis, and clinical management of breast cancer. However, it requires intravenous injection of Gadolinium-contrast agent that entails some elevated risk [16]. Breast MRI is frequently used in the management of breast cancer, especially to determine disease extent in the breast and to direct local therapy. Another promising technique that has garnered substantial interest recently is proton magnetic resonance spectroscopy (1H-MRS). This technique allows quantitative characterization of total or composite choline concentration that has been shown to be elevated in malignant tumors compared to normal breast tissue [17]. Breast MRI will play a role, perhaps complementary to mammography, in screening for high-risk patients. The development of newer and, possibly, more targeted MRI contrast agents may expand the capabilities of breast MRI.

### 2.4. Radionuclide Imaging

Radionuclide-based imaging techniques such as scintimammography, single-photon emission computed tomography (SPECT), positron emission mammography (PEM), and positron emission tomography (PET) are additional imaging techniques that provide for physiologic information. Scintimammography and SPECT typically use 99mTc-Sestamibi or 99mTc-Tetrofosmin for breast-cancer imaging. Scintimammography is used for imaging mostly palpable lesions that were occult or indeterminate from other imaging modalities. Radionuclide imaging has long been used in breast cancer management, primarily in the form of bone scintigraphy (“bone scan”) to detect bone metastases.

18F-fluorodeoxyglucose (FDG) PET is increasingly used in staging advanced or recurrent breast cancer and in monitoring response to therapy; in fact, has received approval for Medicare reimbursement for these clinical indications. For detection of primary tumors, 18F-FDG-PET has been reported to have similar sensitivity as SPECT [18]. A meta-analysis of whole body 18F-FDG-PET that included 13 studies indicated an overall sensitivity of 89% and specificity of 80% [19]. To overcome the limited sensitivity and spatial resolution of whole-body PET systems, a PEM system was developed [20]. The biochemical information provided by radiotracer imaging provides an early window to identify response to systemic therapy and may provide a quantitative endpoint for both clinical practice and clinical trials.

### 2.5. Other Modalities

#### 2.5.1. Volumetric X-Ray Imaging Techniques

 There is a need to develop techniques that provide depth information in breast X-ray imaging. Currently, stereoscopic digital mammography (SDM), digital breast tomosynthesis (DBT), and dedicated breast computed tomography (BCT) are three modalities that are being actively investigated.

#### 2.5.2. Stereoscopic Digital Mammography

In stereoscopic digital mammography, two projection images, spaced a few degrees apart, are acquired with a digital mammography system. A dedicated stereoscopic workstation displays orthogonally polarized images and are viewed by the observer using passive cross-polarized glasses, so that each eye visualizes one image to provide depth perception.

#### 2.5.3. Optical Imaging

Optical imaging techniques of the breast such as diffuse optical tomography (DOT), diffuse optical imaging, and diffuse optical spectroscopy are being investigated as an adjunct technique. Electrical impedance spectroscopy (EIS) and microwave imaging spectroscopy (MIS) are also being explored for potential use in breast cancer detection. 

## 3. Optical Imaging

Optical characterization of the breast has been attempted since 1929 [21] when the term diaphanography was applied to shadowgraphs of breast tissue: *“…a simple procedure and a valuable aid in the interpretation of pathological conditions in the mammary gland. Its use is recommended in the routine examination of the breast…*” [21]. Cutler hoped to distinguish between solid tumors and cysts in the breast, but found it difficult to produce the necessary light intensity for diaphanography without exposing the patient's skin to extreme heat. Although large, highly vascular, malignant lesions could be detected, the method did not achieve sufficient sensitivity and specificity to be used in clinical practice at the time. Transillumination proved largely inadequate for clinical use because it was too difficult to separate the effects of absorption and scattering within the tissue and because the two-dimensional data was poorly suited for image reconstruction. 

Optical imaging as an adjunct device to mammography or ultrasound imaging is a natural choice, because the information gained with the optical signal is markedly different from that of the clinical imaging information. As a result of numerous scientific and technological advances in tissue optics since 1990, optical mammography now appears feasible with levels of specificity and resolution superior to early developments. 

Optical breast imaging is a novel imaging technique that uses near-infrared (NIR) light to assess optical properties of tissues, and is expected to play an important role in breast cancer detection. When fluorescent probes are excited by NIR light, they emit photons at predefined wavelength ranges, detectable by an optical imaging system.

**Figure 1:**
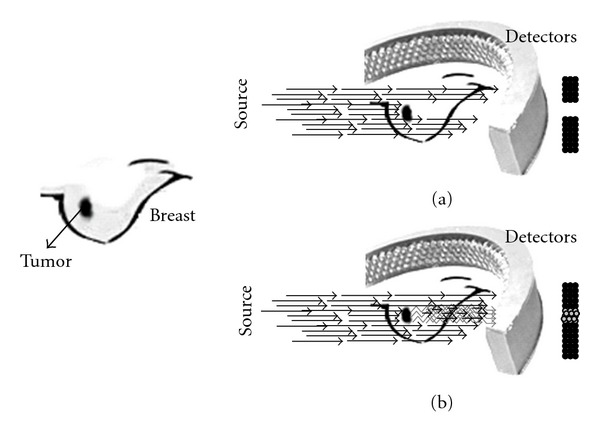
Optical breast-imaging basis. (a) Optical imaging without contrast agent where absorption results in decreased light intensity. (b) Optical imaging with contrast agent where a fluorescent probe emits light at a higher wavelength.

Until now, studies have focused on using the intrinsic optical properties of the breast to visualize lesions without the use of fluorescent contrast agents. These studies described higher absorption for carcinomas than for the surrounding parenchyma due to increased blood content associated with angiogenesis [22–25]. However, intrinsic contrast alone is probably not sensitive enough for (early) lesion detection [26]. Optical breast imaging using a fluorescent contrast agent may improve lesion contrast and can potentially detect changes in breast tissue earlier. The fluorescent probes can either binds specifically to certain targets associated with cancer or can nonspecifically accumulate at the tumor site, mostly by extravasations through leaky vessels (Figure 1).

**Figure 2:**
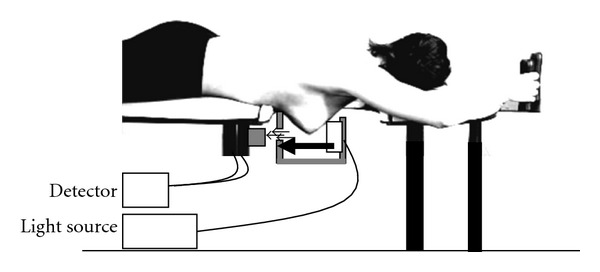
Breast optical imaging prototype. Patient lies in prone position. Soft compression in a plane and detection in the opposite one.

Clinical optical imaging diagnosis started with the development of clinical optical breast imaging systems (Figure 2). Companies and academia put a lot of effort in this task; some systems are commercially available at the moment. The computed tomography laser mammography system CTLM, developed by Imaging Diagnostic Systems Inc., is a fully tomographic system and generates volumetric images of the breast. Poellinger et al. [27] and Floery et al. [28] concluded that CTLM could be used for the delineation of malignant tissue. The ComfortScan system, distributed by Danum International Ltd., is a transillumination system that requires breast compression to generate 2D images. Fournier and colleagues concluded that the system had the potential to distinguish benign from malignant lesions assuming a higher number of false-positive results compared to conventional mammography [29]. The SoftScan system by Advanced Research Technologies Inc., is a system that requires slight breast compression but is able to generate tomographic images of the breast. Using this system, van de Ven concluded that the use of such contrast agents, at least in addition to a targeting ligand, would have a great potential in future optical breast cancer diagnosis [30].

Major advantages of optical imaging are that it does not use any radioactive components (as in PET and SPECT), which can result in repeated use even in young women, and that its sensitivity is very high (nanomolar to picomolar concentration range) compared to MRI, it is relatively inexpensive, and is easily accessible. However, this technique is still in a very early phase of development. 

## 4. Basic Concepts of Optical Breast Imaging

Optical imaging uses light propagation through tissue to assess its optical properties. In general, optical imaging devices transmit light through the breast, where it is both absorbed and scattered by the tissue components present. The light used in optical imaging is commonly monochromic and in the near-infrared (NIR) range permitting imaging up to several centimeters deep in soft tissue. Different tissue components have unique scattering and absorption characteristics for each wavelength. NIR in the wavelength range of 600–1000 nm is used to allow for sufficient tissue penetration. After passing through the breast, the remaining light is registered by detectors and advanced computer algorithms are used to reconstruct the images [31, 32]. 

All optical imaging systems, in general, use three different illumination methods: continuous wave (CW), time-domain photon migration (TDPM), and frequency-domain photon migration (FDPM).

### 4.1. Continuous-Wave (CW) Imaging

Continuous-wave systems emit light at constant intensity or modulated at low frequencies (0.1–100 kilohertz) [33]. The constant intensity source is focused on the tissue, surface, and the tissue volume is illuminated with light whose intensity becomes exponentially attenuated with distance form the surface. It is a straightforward technique, which basically measures the attenuation of light transmitted between two points on the breast surface. Because of its simplicity, continuous-wave equipment is cheap and image acquisition fast.

### 4.2. Time-Domain Photon Migration (TDPM) Imaging

The time domain technique uses short (50–400 picoseconds) light pulses to assess the temporal distribution of photons [34]. Because scattering increases the times of flight spent by photons migrating in tissues, the photons that arrive earliest at the detector have encountered the fewest scattering events. Consequently, a spatial intensity image of early-arriving photons can conceivably be used to detect tissue regions of high absorbance based on their attenuation.

### 4.3. Frequency-Domain Photon Migration (FDPM) Imaging

Frequency domain devices modulate the amplitude of the light that is continuously transmitted at high frequencies (10 MHz–1 GHz). Measuring photons phase-shifts and their amplitude decay (compared to a reference signal), information on optical properties of tissue is acquired and scattering and absorption can be distinguished. When photon density waves encounter tissue regions of varying optical properties, they refract, scatter, and interfere. The result is a “perturbation” whose magnitude is dependent on the location, size, and optical contrast of variations within a tissue volume.

During the last decade, progress in source and detector technology, light-propagation modeling, and potential fluorescent contrast agents, has resulted in a renewed interest in optical imaging [35].

### 4.4. Light Sources

Three types of light sources are used for optical imaging applications: white light, light-emitting diodes (LED), and laser diodes. The spectrum of most white light sources extends over the visible range and into the near-infrared. In light-emitting diodes, free electrons move across a diode junction. In this process, photons are generated. The photon energy is set by the energy drop between the conduction band and the valence band. In laser diodes (or diode lasers), a population inversion is induced. Laser diodes are used widely for optical imaging applications. 

### 4.5. Detectors

*Photodiode* is a robust and inexpensive detector for relatively high light levels. Light with energy greater than band-gap energy hits the photodiode, excites electrons into the conduction band, and creates a hole in the valence band. *Avalanche photodiode (APD)* is a high-speed, high-sensitive photodiode with an internal gain mechanism through a reverse-bias voltage. The electron-hole pairs are generated from exposure to light with higher photon energy than band-gap energy. *Photomultiplier tube (PMT)* is a sensitive detector which amplifies the input light signal 105 to 106 times with almost no additional noise. It is usually selected for high-speed or low-light level detection. It is suitable for single-photon counting applications when the rate photons striking the photocathode are below 100 MHz. *Charge-coupled device (CCD)* is a solid-state sensor with a wafer of silicon crystal. When the silicon is exposed to light, the photoelectric effect generates electrons from the silicon bonds. These free electrons are collected by CCD electrodes at the interface created by positive surface potential to form an extremely thin, but very dense, inversion layer. *Image intensifier* is a vacuum tube device consisting of a photocathode input, microchannel plate, and the phosphor screen. The electrons due to photoelectric effect are accelerated and multiplied through microchannel plate (MCP) via mechanism analogous to photomultiplier, to the phosphor screen where the light is released upon striking the coating.

**Figure 3:**
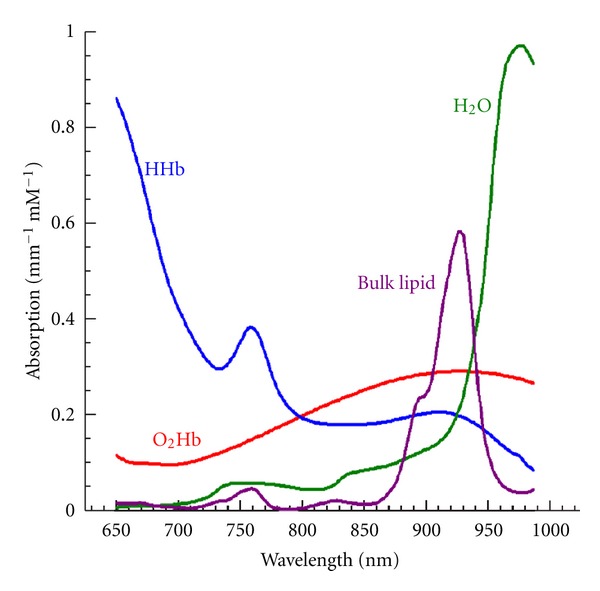
Endogenous absorption related to wavelength. HHb. Hemoglobin. O_2_Hb: Oxigenated Hemoglobin.

Optical breast imaging can be performed (1) relying on intrinsic breast tissue contrast alone (mapping hemoglobin, water, and lipid content (Figure 3)); or (2) with the use of exogenous fluorescent probes that target molecules specific for breast cancer (Figure 1). The use of fluorescent probes has great potential in early breast cancer detection, since in vivo imaging of molecular changes associated with breast cancer formation is technically feasible. 

## 5. Optical Breast Imaging without Contrast Agent

Optical breast imaging uses NIR light to assess the optical properties of breast tissue. Light absorption at these wavelengths is minimal, allowing for sufficient tissue penetration (up to 15 cm). The main components of the breast all have specific absorption characteristics as a function of the wavelength. Imaging of scattering may be associated with structural characteristics and the concentrations of organelles. By combining images acquired at various wavelengths (spectroscopy) concentrations of oxy- and deoxy-hemoglobin, water, and lipid can be determined. In a malignant tumor, hemoglobin concentration is directly related to angiogenesis, the key factor required for tumor growth and metastases [36]. In addition, the proportions of oxy- and deoxy-hemoglobin change in such a tumor due to its metabolism [37]. By measuring concentrations of the breast components, discrimination of benign and malignant tumors may be possible with diffuse optical imaging. More importantly, imaging of the absorption coefficient, at appropriately selected wavelengths, can quantify the concentrations of water and oxy- and deoxy-hemoglobin of breast tumors, and obtain measures of hemoglobin concentration and hypoxia.

The scattering properties of tissues also contain important information for lesion diagnosis. The scattering coefficients are related to the tissue structure properties and the concentration or size of organelles. The *in vivo* measurements show that scattering coefficients are wavelength-dependent. While *ex vivo* studies suggest that the scattering coefficients alone do not provide sufficient information to discriminate the small breast tumors from normal breast tissue (Figure 1). 

Detection rates for the transillumination approach range from 0.58 to 0.94 [34, 38]. With tomography, carcinomas were detected between 50% and 74% [39]. Detection rates of benign lesions vary between 0.04 and 0.94 [34, 38, 39]. Benign cysts were detected with tomography in 83% [40]. Malignant lesions were detected by their higher optical attenuation compared to the surrounding tissue, mainly related to increased light absorption by their higher hemoglobin content [41]. Solid benign lesions were more difficult to detect, but sometimes showed increased attenuation, although to a lesser extent than malignant lesions [34, 38–41].

Optical imaging can probe the concentrations of those chromophores, especially the oxygenated and deoxygenated hemoglobin. Hence, it can provide the biochemical specificity in breast cancer diagnosis. Typically, two important parameters are given by optical spectroscopy and imaging, the blood volume (the sum of the oxygenated and deoxygenated hemoglobin concentration) and the oxygen saturation (the ratio of oxygenated hemoglobin to the blood volume). Statistical data indicate that there are two-to fourfolds of contrast between normal and tumor structures for the blood volume, and the oxygen saturation in the tumor is also less than normal.

## 6. Optical Breast Imaging with Contrast Agent

A novel element that can enhance the potential applications of optical imaging is the use of contrast agents. In optical breast imaging with contrast agent, fluorescent probes are used that emit photons at predefined wavelengths after excitation by laser light. These photons are detected while the light of the excitation wavelength is filtered (Figure 1). Fluorescent probes that target molecules specific for breast cancer are currently being developed and validated in preclinical animal studies [42–45]. New “Smart” optical probes to target proteases are being analyzed [42, 43]. These probes are nonfluorescent in their native state, but convert to a highly fluorescent active state when their backbone is cleaved by cathepsins.

Like other imaging modalities, contrast enhancement through contrast agents, either for absorption or fluorescence, have shown great promise for improving the sensitivity and specificity of breast cancer detection [46].

### 6.1. Nonspecific Contrast Agents

**Figure 4:**
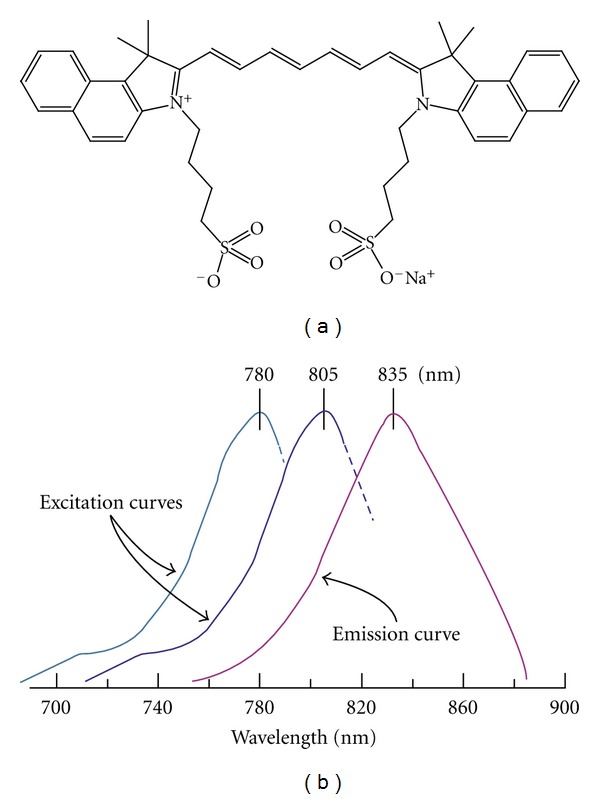
(a) Estructure of ICG. (b) Absorption/fluorescene spectrum of ICG.

The most commonly used contrast agent in the NIR spectral window is Indocyanine Green (ICG) (Figure 4).

ICG is a nonspecific blood pool agent that is both absorbing and fluorescent in the NIR range. It is used clinically, mainly for retinal angiography and liver function tests. Both studies observed differences in ICG pharmacokinetics between malignant and benign lesions on the optical images.

Important advantages of optical imaging with contrast agent are that it does not use any radioactive components (as in PET and SPECT), and that its sensitivity for probe detection is very high (possibly in the nanomolar to the 100 picomolar concentration range) as compared to MRI (micromolar to millimolar range). Moreover, optical imaging uses no ionizing radiation and can thus be used repeatedly, also in younger women. Similar to nuclear imaging, however, the optical method generally can detect very small concentrations of chromophores or fluorophores, but without using ionizing radiation and at a reduced cost.

Few studies reported on optical breast imaging with the use of a fluorescent contrast agent. Two case reports described their experiences using the nonspecific agent Indocyanine Green (ICG), the only fluorescente agent approved for use in humans today [46–48]. Both groups observed a marked absorption increase in the malignant tumors due to accumulation of ICG. In the study by Corlu et al. it was shown that the use of ICG is feasible for fluorescence Diffuse Optical Tomography (DOT). This was the first, and thus far the only, study to demonstrate fluorescence DOT in vivo in three women with breast cancer [49] (Table 1).

### 6.2. Molecular Specific Contrast Agents

Cancer cells overexpress certain receptors, and increase the uptake of the corresponding ligands. This process will result in the accumulation of those ligands in a certain type of cells, thus providing high detection specificity. Conjugation of a fluorophore to those ligands can provide high fluorescent contrast for tumor versus normal cells.

Breast cancer (BC) is a heterogeneous class of disease exhibiting a variety of phenotypes and molecular profiles. For selection of the most promising therapy, with regard to the molecular profile of a cancer lesion, immunohistochemistry (e.g., biopsies) is performed. In this context, identification of for example, hormone receptor positive (estrogens, ER+, progesterone PR+) or human epidermal growth factor receptor 2 positive (HER-2+) cancers are important and significant points to be addressed.

A new advance that holds great promise for breast cancer research is the recent development of optical probes for molecular imaging, specifically in the NIR range. Fluorescent dyes that target specific tumor receptors [50], or that are activated (fluoresce) by tumor-associated enzymes (such as cathepsins and matrix metalloproteases) [51], have been shown to identify their molecular targets *in vivo*. 

Using this technology, appropriately engineered fluorescent probes can be selectively activated by endogenous or transferred gene expression. The combination of such probes with optical imaging may yield a unique, highly sensitive technology for *in vivo* and real-time imaging of the expression patterns for various enzymes, which are crucially involved in tumor formation and metastasis. 

Therefore, the impact of developing molecular-optical imaging, and in particular molecular-DOT, of the breast is potentially enormous. First, selected molecular activity can be achieved with high sensitivity. Second, cancers could be detected at their molecular onset, before anatomic changes become apparent. Therefore, therapies can be initiated at a very early stage. Third, specific cancer parameters such as growth kinetics, angiogenesis growth factors, tumor cell markers, and genetic alterations could be studied without perturbing the tumor environment. Finally, those points are important in the development of novel targeted drugs and therapies. 

## 7. Multimodality Imaging

Another exciting application of optical technology is the combination of optical imaging with other imaging modalities. Light guidance using optical fibers makes optical imaging compatible with many other radiologic methods [52], such as mammography, ultrasound, MRI, and positron emission tomography, among others. The development of hybrid modalities offers the potential of simultaneously scanning of the breast, under identical physiologic conditions. The optical method offers several complementary features to those of established medical imaging methods, mainly through targeting oxy- and deoxy-hemoglobin (Figure 3), but also through the study of molecular events and gene expression. This can produce an increased number of features that may augment the diagnostic value of any single technique alone. 

## 8. Optical-Imaging-Guided Surgery

The only two imaging techniques used, even occasionally, during oncologic surgery are X-ray fluoroscopy (for angiography) and US (for mass detection). However, the former exposes patients and professionals to ionizing radiation, and, the latter requires direct contact with tissue, and neither can be used with targeted contrast agents. Optical imaging, that exploits invisible NIR fluorescent light (700 to 900 nm), offers several advantages for image-guided surgery, including low inherent autofluorescence background, highly sensitive and specific detection of tumors up to millimetres deep in scattering tissue, and real-time imaging (reviewed in Frangioni [53]). Intraoperative imaging systems developed also provide simultaneous acquisition of surgical anatomy (colour video) and function (NIR fluorescence) [54]. Innovative work from other groups has extended depth penetration to several centimetres using frequency domain photon migration techniques. Currently, the only clinically available NIR fluorophore is indocyanine green (Figure 4, Table 1), which is a nontargeted extracellular fluid agent approved for nonfluorescence indications. ICG can be used for NIR fluorescent sentinel lymph node mapping of virtually any tissue or organ [54–56]. Many investigators are also developing NIR fluorophores targeted specifically to human cancer and normal structures [57, 58]. If translated to the clinic, these targeted NIR fluorescent contrast agents would permit the oncologic surgeon to resect malignant cells under direct visualization, while actively avoiding critical structures such as vessels and nerves.

## 9. Conclusions

Optical imaging is a very promising imaging technique. Optical imaging systems can detect imaging agents in picomolar to nanomolar concentration ranges, whereas, for instance, magnetic resonance imaging (MRI) needs larger molecular masses before probe detection is possible (at least micromolar concentrations). In contrast to X-ray, computed tomography (CT) and positron emission tomography (PET), optical imaging uses no ionizing radiation. Repeated imaging is therefore possible without radiation risks. Moreover, optical imaging is relatively cheap, and imaging agents are easy to generate and have long half-lives, particularly compared to PET tracers. 

Main challenges in optical imaging are depth penetration, signal quantification, and development, validation and approval of relevant imaging agents for human use. Light penetration in tissue is limited, but with the use of near-infrared light, and the development of more sensitive detection equipment, penetration in human tissue is now possible up to 15 centimeters deep. 

Another way to deal with the lower spatial resolution is a multimodality approach. Optical imaging could be fused with other anatomical imaging techniques, such as ultrasound (photoacoustic imaging) or MRI, similar to nuclear imaging techniques as PET/CT. 

The other key challenge in clinical translation of optical imaging is the development of relevant imaging agents. Crucial for this step is the full understanding of the molecular biology of breast cancer to identify potential targets. Biologic processes to study for target identification include, for example, tumor metabolism, angiogenesis, proliferation, apoptosis, and hypoxia.

 When clinical translation of appropriate optical imaging agents will be successful, optical breast imaging could improve early detection of breast cancer; for example, in women with dense breasts who are at increased risk for breast cancer. X-ray mammographic screening has very limited sensitivity in these women due to the tumor-hiding projection of the dense glandular tissue [59, 60], while near-infrared light is far less hindered by dense breast tissue. Optical breast imaging could also have a role in the selection of appropriate adjuvant treatment, the evaluation of response to treatment, and the fine-tuning of treatment strategy in the individual breast cancer patient. Moreover, optical imaging agents could, potentially, be used as “theranostics”, combining the process of diagnosis and (local) therapy [61]. 

## Figures and Tables

**Table 1:** Devices used for clinical trials of ICG Fluorescence imaging. adapted from: Marshal et al.; The Open Surgical Oncology Journal, 2010, 2, 12–25.

Device	Excitation source	Fluorescence collection	Detector	Working distance	Field of view	Depth of penetration	Integration time or frames per Sec (FPS)
Photodynamic Eye (PDE) Hamamatsu	Laser-emitting diodes (LEDs) centered at 760 nm, incident power not specified	Bandpass filter > 820 nm	CCD	20 cm	Not given, but limited	2 cm	Not specified
SPY (Novadaq)	Laser-emitting at 806 nm, 2.0–2.7 W, incident power not specified	835 nm “camera,” not specified	CCD	30 cm	56 cm^2^	1 mm DOP	30 fps
FDPM imager (Texas)	Laser diode, 785 ± 10 nm. <1.9 mW/cm^2^	Notch filters at 785 nm, and at 830 nm	Gen III intensifier coupled to CCD, gain modulatable for tomography	Variable, but reported <76.2 cm	Max reported FOV 900 cm^2^	Estimated to be 4 cm	50–800 msec
IC-View (Pulsion Medical)	Laser diode 780 nm (0.16 W), incident power not specified	Not specified	CCD	Not specified	Not specified	Not specified	Not specified
FLARE (Israel Beth Deaconess Hospital)	LEDs emitting 745–779 nm, 14 mW/cm^2^	Bandpass filter 800–848 nm	CCD	45 cm	3.7 cm^2^–169.5 cm^2^	Not specified	200 msec
Custom system (Kochi Medical School)	LEDs emitting light centered at 760 nm, incident power not specified	840 nm cut-on filter	Color CCD	~50 cm	78.5 cm^2^	Not specified	Not specified
